# Establishing a trigger tool based on global trigger tools to identify adverse drug events in obstetric inpatients in China

**DOI:** 10.1186/s12913-023-10449-z

**Published:** 2024-01-15

**Authors:** Shan Wu, Qinan Yin, Liuyun Wu, Yue Wu, Nan Yu, Junfeng Yan, Yuan Bian

**Affiliations:** 1 Department of Pharmacy, Sichuan Provincial People’s Hospital, School of Medicine, University of Electronic Science and Technology of China, Chengdu, China; 2 Maternal and Child Health Hospital of Shuangliu District, Chengdu, China; 3 Personalized Drug Therapy Key Laboratory of Sichuan Province, School of Medicine, University of Electronic Science and Technology of China, Chengdu, China; 4 Chengdu First People’s Hospital, Chengdu, China

**Keywords:** Trigger tool, Global trigger tool, Obstetric department, Adverse drug event

## Abstract

**Background:**

Pregnant women belong to the special population of drug therapy, and their physiological state, pharmacokinetics and pharmacodynamics are significantly different from the general population. Drug safety during pregnancy involves two generations, which is a hot issue widely concerned in the whole society. **Global Trigger Tool (GTT) of the Institute for Healthcare Improvement (IHI)** has been wildly used as a patient safety measurement strategy by several institutions and national programs, and the effectiveness had been demonstrated. But only one study reports the use of GTT in obstetric delivery until now. The aim of the study is to establish triggers detecting adverse drug events (ADEs) suitable for obstetric inpatients on the basis of the GTT, to examine the performance of the obstetric triggers in detecting ADEs experienced by obstetric units compared with the spontaneous reporting system and GTT, and to assess the utility and value of the obstetric trigger tool in identifying ADEs of obstetric inpatients.

**Methods:**

Based on a literature review searched in PubMed and CNKI from January of 1997 to October of 2023, retrospective local obstetric ADEs investigations, relevant obstetric guidelines and the common adverse reactions of obstetric therapeutic drugs were involved to establish the initial obstetric triggers. According to the Delphi method, two rounds of expert questionnaire survey were conducted among 16 obstetric and neonatological physicians and pharmacists until an agreement was reached. A retrospective study was conducted to identity ADEs in 300 obstetric inpatient records at the Sichuan Academy of Medical Sciences & Sichuan Provincial People’s Hospital from June 1 to September 30, 2018. Two trained junior pharmacists analyzed the first eligible records independently, and the included records reviewed by trained pharmacist and physician to identify ADEs. Sensitivity and specificity of the established obstetric triggers were assessed by the number of ADEs/100 patients and positive predictive value with the spontaneous reporting system (SRS) and GTT. Excel 2010 and SPSS22 were used for data analysis.

**Results:**

Through two rounds of expert investigation, 39 preliminary triggers were established that comprised four modules (12 laboratory tests, 9 medications, 14 symptoms, and 4 outcomes). A total of 300 medical records were reviewed through the obstetric triggers, of which 48 cases of ADEs were detected, with an incidence of ADEs of 16%. Among the 39 obstetric triggers, 22 (56.41%) were positive and 11 of them detected ADEs. The positive predictive value (PPV) was 36.36%, and the number of ADEs/100 patients was 16.33 (95% CI, 4.19–17.81). The ADE detection rate, positive trigger rate, and PPV for the obstetric triggers were significantly augmented, confirming that the obstetric triggers were more specific and sensitive than SRS and GTT.

**Conclusion:**

The obstetric triggers were proven to be sensitive and specific in the active monitoring of ADE for obstetric inpatients, which might serve as a reference for ADE detection of obstetric inpatients at medical institutions.

**Supplementary Information:**

The online version contains supplementary material available at 10.1186/s12913-023-10449-z.

## Introduction

Adverse drug event (ADE) is an injury caused by drug-related medical interventions, including non-preventable adverse drug reactions (ADRs) and preventable medication errors [1, 2]. As one of the leading causes of deaths, hospitalizations, and increased treatment costs [3–7], the effective identification and supervision of ADEs has become a major concern of scientific research and health management departments. The fact that most ADEs are preventable makes the monitoring and reporting of ADEs particularly important [8, 9]. The Global Trigger Tool (GTT), an active monitoring tool launched by the Institute for Health Care Improvement (IHI) in 2003 and revised in 2009, is able to detect medically related adverse events with six modules of “nursing”, “medication”, “surgical”, “intensive care”, “perinatal”, and “emergency” [10]. Compared with the spontaneous reporting system (SRS) and adverse event medical record review, GTT purposefully locates ADE-related content, thereby improving the efficiency and accuracy of case review [11]. Numerous studies have validated the accuracy and effectiveness of GTT in ADE monitoring. The translation and revision of the GTT White Paper are established to adapt to national circumstances, study population and healthcare facility. However, studies in special populations have mainly focused on elderly, pediatric, cancer, and intensive care unit (ICU) inpatients, and only one study has explored the applicability of GTT in obstetric populations [12–21].

Chinese society is currently dealing with challenges of declining and delaying fertility intentions of women at childbearing age, increasing infertility rates and aging of maternal [22]. The risk of maternal and infant exposure to medication during pregnancy increased alongside with the incidence of pregnancy complications [23, 24]. In addition, there are significant changes in the pharmacokinetic profile of pregnant population [25, 26]. A study in France [27] showed that ADRs were more common in pregnant patients than in non-pregnant patients. Among 53,426 ADRs documented in Sichuan Province between November 2016 and November 2017, a mere 1309 ADRs pertained to pregnant patients, constituting a mere 2.45% of the total [28]. In 2016, the International Network for Rational Use of Drugs (INRUD) / China Center Clinical Safety Medication Group recorded a total of 84 medication errors involving pregnant and lactating patients, accounting for 1.27% of the 6624 reported nationwide medication errors [29]. The limited efficiency of prevailing reporting methodologies, coupled with the few information-reporting members within INRUD China, particularly within women’s and children’s specialty hospitals, contributed to the relatively diminutive number of reported medication errors. As a result, ADEs in obstetric patients may has been potentially underestimated. In this study we devised a novel trigger tool with high-efficiency, leveraging the GTT which can be implemented to identify ADEs in obstetric inpatients retrospectively. Based on detectable results, the trigger tool could then be modified to align with Chinese obstetric inpatients.

## Methods

### Literature search

We systematically reviewed the literature spanning from January 1997 to October 2023, utilizing PubMed and CNKI database, employing the keywords “gestational trigger tool”, “trigger tool”, “gestational”, “obstetric”, “trigger tool”, “obstetrics”, and “pregnancy”. Our inclusion criteria comprised (1) specific trigger entries; (2) application of triggers in obstetric patients experiencing ADEs; and (3) incorporation of detection results. Upon scrutinizing the abstracts and results of the literature obtained through the search, we found that as of October 2023, only one GTT-based study was conducted in a maternal population, using the 44 triggers of the Swedish adaption and translation of GTT [21]. Therefore, triggers applied to general adult inpatients were included.

### Trigger extraction and revision

Preliminary triggers were extracted from the included literature. Subsequently, guided by obstetric guidelines, ADEs among obstetric patients documented in the Chinese National Adverse Drug Reaction Monitoring System (NADRMS), prevalent ADEs associated with pharmaceutical interventions for special obstetric conditions, and in sight from the Williams Handbook of Obstetrics, our results underwent comprehensive evaluation by a review panel composed of pharmacists and physicians.

### Delphi experts investigation

The Delphi method [30] was employed to administer an expert survey within the scope of this study. A cohort of 16 comprising obstetricians, neonatologists and pharmacists was randomly selected from healthcare facilities nationwide, following a process of informed and voluntary basis. The initial set of triggers underwent modifications based on expert recommendations, encompassing the rationale and interpretation of entry parameters. Following two rounds of revisions, triggers exhibiting high consistency among experts were retained.

### Retrospective records review

The study was undertaken following the approval of the ethical review committee at Sichuan Academy of Medical Sciences & Sichuan Provincial People’s Hospital. A total of 300 discharged medical records from the aforementioned hospital, pertaining to the third quarter of 2018, was selected through a random sampling process. The specified inclusion criteria encompassed (1) medical records discharged between July 1, 2018, and September 30, 2018; (2) obstetric inpatients with a gestational age of ≥ 28 weeks; (3) individuals aged between 16 and 65 years; and (4) patients with a hospitalization duration exceeding 48 h. Exclusion criteria were applied to (1) cases lacking treatment-related medication records; and (2) instances where essential primary data from the inpatient medical records were absent.

Our review panel was instituted in accordance with the guidelines outlined in the IHI white paper. Initial scrutiny of the foundational obstetric triggers was conducted by two junior pharmacists, followed by a comprehensive review by a senior pharmacist and a physician. Based upon the records, the panel appraised the presence of a positive trigger and ascertained the occurrence of an ADE, arriving at a consensus on these matter. The causality of the ADE was assessed according to the World Health Organization-Uppsala Monitoring Center (WHO-UMC) standards, including certain, probable/likely, possible, unlikely, conditional/unclassified, and inaccessible/unclassifiable as shown in Table 1. Obstetricians and pharmacists conducted a comprehensive review of triggered items, judged symptoms based on the WHO-UMC causality categories, and categorized specific medical records (certain, probable/likely medical records) in the category of ADEs [31]. In accordance with Common Terminology Criteria for Adverse Events (CTCAE) 5.0, the severity of the ADE injuries was stratified into five levels [32], as presented in Table 2. Flowchart of the study sample process and medical record review sheet for the application of the obstetric trigger tool are accessible in supplementary documents.

**Table 1:** WHO-UMC causality categories

Causality term	Assessment criteria
Certain	• Event or laboratory test abnormality, with plausible time relationship to drug intake • Cannot be explained by disease or other drugs • Response to withdrawal plausible (pharmacologically, pathologically) • Event definitive pharmacologically or phenomenologically (i.e., an objective and specific medical disorder or a recognized pharmacological phenomenon) • Rechallenge satisfactory, if necessary
Probable / Likely	• Event or laboratory test abnormality, with reasonable time relationship to drug intake • Unlikely to be attributed to disease or other drugs • Response to withdrawal clinically reasonable • Rechallenge not required
Possible	• Event or laboratory test abnormality, with reasonable time relationship to drug intake • Could also be explained by disease or other drugs • information on drug withdrawal may be lacking or unclear
Unlikely	• Event or laboratory test abnormality, with a time to drug intake that makes a relationship improbable (but not impossible) • Disease or other drugs provide plausible explanations
Conditional / Unclassified	• Event or laboratory test abnormality • More data for proper assessment needed, or additional data under examination
Unassessable / Unclassifiable	• Report suggesting an adverse reaction • Cannot be judged because information is insufficient or contradictory • Data cannot be supplemented or verified

**Table 2:** CTCAE5.0 General Guideline

Grade 1	Mild; asymptomatic or mild symptoms; clinical or diagnostic observations only; intervention not indicated
Grade 2	Moderate; minimal, local or noninvasive intervention indicated; limiting age-appropriate instrumental ADL*
Grade 3	Severe or medically significant but not immediately life-threatening; hospitalization or prolongation of hospitalization indicated; Disabling; limiting self-care ADL**
Grade 4	Life-threatening consequences; urgent intervention indicated
Grade 5	Death related to AE.

ADL is short for Activities of Daily Living

* Instrumental ADL, refers to preparing meals, shopping for groceries or clothes, using thetelephone, managing money, etc

** Self-care ADL, refers to bathing, dressing and undressing, feeding self, using the toilet, taking medications, and not bedridden

The causality determination and severity classification were compared with the GTT and SRS to scrutinize and substantiate the effectiveness of the formulated obstetric triggers. Subsequently, the triggers underwent revision based on the outcomes of the review.

Sensitivity assessment of the triggers was conducted through adverse events per 100 admissions, adverse events per 1000 patient days, and ADE detection rate; while specificity was appraised utilizing trigger PPV analysis (ADE detection frequency/trigger-positive trigger frequency).

### Statistical method

Excel 2010 and SPSS 22.0 were used to analyze the data with descriptive statistics displaying frequencies, percentages, means and standard deviations. Regression analysis was performed to determine the correlation of the variables. Statistical significance was established when the *P*-value fell below 0.05.

## Results

### Trigger extraction and revision

We included 43 [4, 8–11, 13, 16, 20, 21, 33–66] articles based on our inclusion criteria, with almost half of them addressing the triggers recommended in the white paper. A total of 41 triggers were identified from various sources, including the articles, the white paper, physiologic changes during pregnancy, the common ADEs of drugs administered to obstetric patients for specific conditions, the Williams Handbook of Obstetrics [67], the obstetric guidelines, and a study of ADEs of obstetric patients [28]. 39 triggers (Table 3) were ultimately defined through two rounds of expert surveys involving modifications to the initially identified 41 triggers, organized into four distinct modules encompassing 12 triggers related to laboratory examinations, 9 related to medications, 14 related to symptoms, and 4 related to outcomes.

**Table 3:** Triggers and PPV

Modules	No.	Triggers	Interpretation	Positive triggers	ADEs	PPV*(95%CI), %
Laboratory tests	L1	K < 3.3mmol/L	hypokalemic drugs used	1	0	0.00%
	L2	K > 5.5mmol/L	hyperkalemic drugs used	0	0	-
	L3	Mg > 3.5mmol/L	hypermagnesemia drugs used	0	0	-
	L4	Na < 130mmol/L	hyponatremic drugs used	0	0	-
	L5	Non-diabetic patients: BG < 3.3mmol/L Diabetic patients receiving hypoglycemic therapy: BG < 3.9mmol/L	hypoglycemic drugs used inappropriately	17	0	0.00%
	L6	Patients receiving hypoglycemic therapy: FBG > 5.3mmol/L, 1 h PBG > 7.8mmol/L, 2 h PBG > 6.7mmol/L Non-diabetic patients: FBG ≥ 6.1mmol/L, PBG ≥ 7.8mmol	hyperglycemic medications used inappropriately	4	0	0.00%
	L7	SCr increased ≥ 0.3 mg/dL within 48 h or SCr increased to ≧ 1.5 times of baseline within seven days or urine volume < 0.5mL/(kg·h) & duration > 6 h	nephrotoxic drugs used	0	0	-
	L8	PT > 13s; APTT > 35s; INR ≥ 1.5; combined with bleeding symptoms	heparin used excessively	0	0	-
	L9	Platelets < 50 × 10^9^/L (excluding physiologic changes and reductions caused by comorbid diseases)	thrombocytopenia drugs used	3	0	0.00%
	L10	①Hyperthyroidism: TSH decreases, TT4 and FT4 increase; ②Hypothyroidism TSH > 4.0mIU/L or TPOAb + and TSH 2.5–4.0mIU/L; Excluding combined thyroid disease	antithyroid drugs/hyperthyroidism drugs used	1	0	0.00%
	L11	WBC Ct < 5.9 × 10^9^/L; neutrophil count < 3900/µL (excluding decreases caused by disease changes)	leukopenia drugs used	2	1	50.00%
	L12	ALT ≥ 3ULN & R ≥ 5, ALP ≥ 2ULN & R ≤ 2, ALT ≥ 3ULN, ALP ≥ 2ULN & 2 < R < 5, R= (ALT measured value/ALTULN) / (ALP measured value/ALPULN)	hepatotoxic drugs used	7	0	0.00%
Medications	M1	protamine given	after heparin administration	0	0	-
	M2	Use of glucocorticoids/antihistamines/calcium gluconate	after drug allergy or anaphylaxis/anaphylactic shock caused by transfusion	1	1	100.00%
	M3	Use of adrenaline	anaphylactic shock	0	0	-
	M4	50% glucose injection (neonates 10%) administered	after drug-induced severe hypoglycemia	0	0	-
	M5	narcan (naloxone)/nalmefene	after opioid poisoning	0	0	-
	M6	laxative or stool softener given	after drug-induced constipation	36	2	5.56%
	M7	Use of live intestinal bacteria preparations/antidiarrheal agents such as montmorillonite	after drug-induced diarrhea	0	0	-
	M8	Use of antiemetics (excluding nausea of pregnancy)	after drug-induced vomiting	1	1	100.00%
	M9	Intravenous injection of calcium gluconate	after magnesium sulfate administration	15	0	0.00%
Symptoms	S1	Skin allergic reaction	after antibiotics/drugs that cause skin reactions administration	3	2	66.67%
	S2	Hypotension/falls	after antihypertensive drugs, sedative hypnotics, and other drug administration	10	6	60.00%
	S3	Elevated blood pressure: higher than systolic blood pressure of 140 mmHg and/or diastolic blood pressure of 90 mmHg (excluding poorly controlled hypertension)	after hypertensive drugs, prostaglandin drugs, ergonovine administration	11	11	100.00%
	S4	Bleeding (including nasal bleeding, gum bleeding, gastrointestinal bleeding, skin purpura)	after drug-induced bleeding (e.g., aspirin)	0	0	-
	S5	Weak contractions, postpartum hemorrhage	after sedatives, analgesics, magnesium sulfate administration	4	3	75.00%
	S6	Excessive uterine contractions, uterine rupture	after oxytocin, prostaglandins, ergonovine administration	7	5	71.43%
	S7	Acral edema, facial edema, periorbital edema, pulmonary edema (the edema is not caused by the original disease)	after hormones, analgesics, NSAIDS, ergonovine administration	0	0	-
	S8	Thromboembolic events (DVT or PE) (excluding spontaneous embolism caused by pregnancy)	①after drugs that may cause thromboembolism ②Insufficient use of anticoagulant drugs	0	0	-
	S9	Basal body temperature rise ≥ 2 °C, high fever, chills (excluding stress and infection factors)	Using drugs that increase body temperature and chills	0	0	-
	S10	Nervous system symptoms (dizziness, headache, facial or extremity numbness, lethargy, and fatigue)	after oxytocin, prostaglandins, antibiotic drugs administration	2	2	100.00%
	S11	Gastrointestinal discomfort such as nausea and vomiting (excluding morning sickness during pregnancy)	after drugs causing adverse gastrointestinal reactions	9	9	100.00%
	S12	Vaginal discomfort (burning sensation, pain, and local bleeding) (excluding vaginal discomfort caused by diseases such as vaginitis)	after topical vaginal medication administration	0	0	-
	S13	Heart rate higher than 140 beats/min, arrhythmia	after oxytocin, prostaglandins, ergonovine administration	4	4	100.00%
	S14	Oligohydramnios or oligohydramnios (premature rupture of membranes excluded)	after oxytocin, prostaglandins, ergonovine administration	0	0	-
Outcome	O1	admission to ICU /rescue	ADE-induced serious illness	2	2	100.00%
	O2	Abrupt cessation of medication (long-term use of anticoagulants, antihypertensives, hypolipidemic, hypoglycemic or hormones)	ADE caused withdrawal or ADE appeared due to withdrawal	3	0	0.00%
	O3	Neonatal asphyxia, fetal distress, premature delivery, and neonatal respiratory depression (excluding neonatal umbilical cord torsion and other diseases)	Use of drugs that adversely affect newborns (analgesics, oxytocin, prostaglandins, and ergonovine)	11	7	63.64%
	O4	Neonatal withdrawal symptoms (neonatal hypoglycemia, hypotension, neonatal bleeding, bradycardia, neonatal abnormal muscle movement, lethargy, severe breathing difficulties, and feeding difficulties	Use of drugs that adversely affect newborns (analgesics, oxytocin, prostaglandins, and ergonovine)	0	0	-

Abbreviations: BG, blood glucose; FBG, fasting blood glucose; PBG, postprandial blood glucose; SCr, serum creatinine; PT, prothrombin time; APTT, activated partial thromboplastin time; INR, international normalized ratio; TSH, thyroid stimulating hormone; WBC, white blood cells; NC, neutrophil count; ALT, alanine aminotransferase; ALP, alkaline phosphatase.

* PPV = ADEs/positive triggers.

- none

### Patient characteristics

According to the inclusion and exclusion criteria, 300 eligible cases were systematically chosen through random selection, with an average age of 27.45 years, ranging from 18 to 43 years. The duration of hospitalization varied between 2 and 10 days, with an average length of stay recorded at 4.34 days. Within the cohort of 300 subjects, there were 115 instances of cesarean section, 162 cases of natural delivery, and 23 occurrences of fetal preservation. Among the latter, 8 cases were related to threatened premature delivery, 11 cases were associated with gestational cholestasis, 3 cases involved fetal growth restriction, and one case manifested abnormal liver enzymes during gestation.

### Triggers

We conducted a comprehensive examination of 300 medical records utilizing aforesaid 39 triggers. Among these, 22 triggers (56.41%) yielded positive results, and 11 of them successfully identified ADEs. In total, 49 ADEs were reviewed, with only one case (0.33%) not triggering any of the designated entries during the evaluation process. Within the cohort of 300 obstetric inpatients, 120 exhibited positive triggers, resulting in a positive rate of 40.00%. Notably, a total of 154 triggers were identified as positive, indicating an average of 1.28 triggers per patient. The frequency of ADE detection amounted to 56 cases, yielding a PPV of 36.36%. The ratio of ADEs per 100 patients was 16.33 (95% CI, 4.19–17.81), while the ADEs per 1000 patients*days was 36.89 (96% CI, 32.72–41.07). The detailed results of trigger monitoring can be found in Table 3.

49 ADE cases were identified through the implementation of obstetric triggers, with the detection rate of 97.96%. Additionally, 9 ADEs were identified using the 13 triggers recommended in the white paper, resulting in a detection rate of 18.37%. Concurrently, 7 ADE cases were reported in the SRS, with a corresponding ADE detection rate of 14.29%. The comparison results of these three methods are presented in Table 4.

**Table 4:** ADE detection status among the three methods

Method	Obstetric Triggers		SRS		GTT		Total
	Positive trigger	No positive trigger	Report	Not reported	Positive trigger	No positive trigger	
ADE detection Number	48	1	7	42	9	40	49
ADE detection rate (95% CI)	97.96% (86.87–109.05%)	2.04% (1.81–2.27%)	14.29% (13.60–14.98%)	85.71% (81.57–89.85%)	18.37% (16.29–20.45%)	81.63% (72.39–90.87%)	100%
ADE occurrences per 100 patients	16 (14.19–17.81)	0.33 (0.30–0.37)	0.43 (0.41–0.45)	2.55 (2.43–2.67)	3 (2.66–3.34)	13.33 (11.82–14.84)	16.33 (14.49–18.18)
ADE occurrences per 1000 patient days	36.89 (32.72–41.07)	0.77 (0.68–0.86)	0.79 (0.75–0.83)	4.75 (4.52–4.98)	6.92 (6.14–7.70)	30.75 (27.27–34.23)	37.66 (33.40–41.93)

As depicted in Table 5, five triggers of the GTT were positive, yielding a positivity rate of 38.46%. 31 of the 300 obstetric inpatients had positive triggers, with a positive rate of 10.33%. Of these, 31 triggers were detected as positive, with an average of one trigger per patient. 10 ADEs were detected and the PPV was 32.26%.

**Table 5:** Trigger conditions for the GTT

No.	Screening index	Positive trigger frequency	ADE detection frequency	PPV
M4	BG < 2.8mmol/L	17	0	0.00%
M7	Diphenhydramine (Benadryl) Administration	1	1	100.00%
M10	Anti-Emetic Administration	1	1	100.00%
M11	Over-Sedation/Hypotension	10	6	60.00%
M12	Abrupt Medication Stop	2	2	100.00%
Total		31	10	32.26%

In comparison to the SRS and the GTT, the obstetric triggers exhibited a notably elevation in the ADE detection rate, positive trigger rate, and PPV value, confirming the specificity and sensitivity of the obstetric triggers.

### Characteristics of ADEs

49 cases of ADE were detected, with an incidence of 16.33%. The detected ADEs contained 10 categories primarily affecting the cardiovascular system (17 cases, 34.69%), gastrointestinal system (12 cases, 24.49%), female reproductive system (eight cases, 16.33%), and the fetus (seven cases, 14.29%).

15 distinct drug types were implicated, with the foremost three being medications for the reproductive system (31 cases, 52.54%), electrolyte drugs (primarily magnesium sulfate injection) (10 cases, 16.95%), and central nervous system drugs (10 cases, 16.95%).

In accordance with the CTCAE5.0, 17 cases of ADEs were categorized as grade 1 (17/49, 34.6%), 27 cases as grade 2, (27/49, 55.10%), and 5 cases as level 3, (5/49, 10.20%). No instances of grade 4 or grade 5 severity were identified.

### Risk factors

In our logistic regression analysis (Table 6), the variables of age, hospitalization duration (in days), the quantity of drugs administered, and whether a cesarean section or vaginal delivery was performed did not demonstrate statistical significance (*P* > 0.05). Conversely, the variable representing the number of administered antimicrobials yielded statistical significance, aligning with previous literature suggesting that antimicrobial medication serves as a risk factor for ADEs [6]. Nevertheless, the regression coefficient β was negative, signifying a negative correlation with the incidence of ADEs, which may be attributed to the influence of the included risk factors in our study and a potentially inadequate sample size. Inadequate sample sizes may result in insufficient representation of ADE occurrences, consequently yielding less representative experimental results. Although the Mevik’s study showed that enlargement in sample size did not markedly increase the type and severity of ADEs, it did enhance the detection rate of ADEs [68]. Boxun Chen’s incorporation of triggers into the information system resulted in a more than fourfold increase in the ADE cases detected after one year compared to the period before the intervention [69]. Thus, for future analyses, it is imperative to expand the sample size to achieve a closer approximation to the actual incidence of ADEs. Additionally, consideration of other potential risk factors is warranted to comprehensively understand the complex dynamics influencing ADE occurrence.

**Table 6:** Logistic regression results of risk factors for the occurrence of ADE

Factor	OR (95% CI)	*P* value
Age	1.059 (0.985–1.138)	0.123
Length of hospital stay	1.153 (0.869–1.3529)	0.324
Number of drugs	1.093 (0.848–1.409)	0.493
Number of antibacterial drugs	0.273(0.094–0.792)	0.017
Number of injection drugs	0.816 (0.511–1.303)	0.394
Cesarean section	1.181 (0.399–3.501)	0.764
Vaginal delivery	0.520 (0.082–3.294)	0.487

## Discussion

### Sample size

We randomly selected 300 medical records for the purpose of our analysis, averaging 50 copies biweekly, surpassing the sample size outlined in the white paper. The Mevik’s [68] study indicated that augmenting the sample size produced no significant effect on the type and severity of ADE detection; however, it did contribute to an elevation in the detection rate of ADEs. In our current study, the ADE detection rate stood at 36.89 ADEs per 1000 patient days, a figure closely aligning with the rate of 39.3 ADEs per 1000 patient days in Mevik’s study [68], where 70 samples were drawn every two weeks (totaling 1680). Nevertheless, the inclusion of the medical records was concentrated in the third quarter, potentially introducing bias into our findings. Consequently, to conduct a comprehensive analysis of risk factors of ADEs in obstetric inpatient and to enhance the ADE detection rate, further expansion of our study’s sample size is warranted.

### ADE detection

A total of 48 ADEs were detected through the established obstetric triggers, and the incidence of ADE was comparable to the findings reported in existing literature, ranging between 10% and 20%. The majority of the detected ADEs, constituting 89.8%, were characterized as mild-to-moderate injuries. Notably, there were no instances of ADEs resulting in permanent injuries or fatalities, which potentially attributed to the limitations imposed by the sample size. The predominant categories of identified ADEs were associated with the cardiovascular system, gastrointestinal system, and female reproductive system, accounting for 34.69%, 24.49%, and 16.33%, respectively. The cardiovascular system injuries were specifically manifested as elevated blood pressure and hypotension. Blood pressures that exceeded 140/90 mmHg were primarily due to the use of oxytocin and ergonovine, while hypotension was caused by magnesium sulfate. The application of prostaglandin drugs led to nausea, vomiting, diarrhea, or other gastrointestinal disturbances and excessive uterine shrinkage. This ADE-detection result was consistent with the clinical medication characteristics of obstetric inpatients and a study on the occurrence of ADRs in local obstetric patients [28].

However, in patients with high-risk pregnancies, the manifestations of complications are very similar to the symptoms of ADE caused by the therapeutic agents, posing challenges in discerning the presence of an ADE. For example, patients experiencing eclampsia and hemolysis, elevated liver enzymes and low platelets syndrome (HELLP syndrome) commonly exhibit symptoms such as headache, nausea and vomiting, which are also common adverse reactions attributed to uterotonics. The new-onset hypertension in the postpartum period may be attributed to postpartum pre-eclampsia, the administration of ergot derivatives for the prevention or treatment of postpartum hemorrhage (PPH), and/or the prolonged administration of high doses of non-steroidal anti-inflammatory drugs (NSAIDs) for postpartum analgesia [70, 71]. The excessive and prolonged utilization of oxytocin, appliance of magnesium sulfateIn and anaesthetics increases the risk of weak uterine contractions leading to PPH, while pre-eclampsia and HELLP syndrome are significantly associated with postpartum haemorrhage [72, 73]. Conditions such as placenta praevia, placental abruption and pre-eclampsia can lead to fetal distress due to diminished utero-placental blood flow, while inappropriate use of uterotonics and intrathecal administration of opioids for labour analgesia can induce tonic uterine contractions, which in turn can lead to fetal distress [74]. Another obvious side effect of misoprostol pertains to its induction of hyperthermia, with the degree of hyperthermia escalating proportionally to the administered misoprostol dosage. A randomized trial including patients with PPH showed that patients receiving 600 µg sublingual misoprostol and a standard contraction agent (contraction in 98% of patients) exhibited a threefold higher incidence of a temperature ≥ 38 °C (58% vs. 19%) compared to patients solely administered a standard contraction agent; the occurrence of a temperature ≥ 40 °C was 7% in the former group and < 1% in the latter [75]. Such cases necessitate a comprehensive evaluation encompassing medical history, physical examination, and laboratory assessments to discern potential pathological effects. The determination of the causal relationship between symptoms and medication will be adjudicated employing the WHO-UMC Causality Categories. Cases posing challenges in identification will be deliberated by a review panel, culminating in a consensus as the ultimate resolution [39].

Through the observation of doctors and nurses in the surgical setting, we ascertained that certain ADEs were unrecorded when the clinical manifestations were mild and did not necessitate specialized treatment. We therefore posit that it is imperative to augment the proficiency of medical personnel in recognizing and summarizing prevalent ADEs in the field of obstetrics.

### Validity of triggers

48 cases of adverse reactions were detected based on the established obstetric triggers, whereas only 7 cases were reported by the SRS within the same timeframe, underscoring the heightened monitoring performance of the obstetric triggers compared to the SRS. This result aligns with the conclusions reached by Classen et al. [4], which revealed that “the detection rate of ADE by trigger tool was about 10 times higher than the previous two methods (SRS and patient safety indicator monitoring).” Through two rounds of the Delphi method in our current study, the established obstetric triggers manifested a PPV of 36.36%, surpassing the sensitivity and specificity of both the SRS and GTT, therefore substantiating the effectiveness of the obstetric triggers. However, among the 39 triggers examined, 17 failed to activate, yielding a negative activation rate of 43.59%. The PPV for 5 triggers was 100%, while the trigger frequency fluctuated between a minimum of one occurrence and a maximum of 11. Thus, given the low positive trigger rates associated with certain triggers, further revision is required.

### Revision of the triggers

A new round of trigger revisions was executed based on the results of the medical records review.

#### Revision of untriggered trigger entries

With respect to the untriggered items, M1 “protamine given”, S4 “bleeding”, S8 “thromboembolic events”, and S14 “oligohydramnios or oligohydramnios” were not triggered. These four entries (M1, S4, S8, and S14) have been omitted in alignment with a drug used during the perinatal period and its trigger probability.

#### Revision of triggers reflecting a low PPV

To improve trigger accuracy, we conducted revisions for triggers exhibiting a low PPV. In the course of reviewing medical records, we observed that the trigger “intravenous injection of calcium gluconate” was triggered 15 times, principally in patients with eclampsia or pre-eclampsia, without any corresponding ADE noted. Studies have shown that Ca^2+^ stimulates neuromuscular excitement, promotes blood coagulation, and, when administered intravenously before cesarean section, diminished oxytocin levels, intraoperative and postoperative bleeding, thereby effectively prevented postpartum hemorrhage [76, 77]. The condition of this trigger entry should therefore be defined as “intravenous injection of calcium gluconate and Mg > 5 mmol/L.”

Among the enrolled patients, 115 underwent cesarean section; and those who did not experience flatulence in the initial two days post-surgery were administered keratin and/or lactulose to ameliorate constipation arising from the surgical procedure. As a result, only 2 ADEs were identified in the 36 triggers categorized under “laxative or stool softener given”. Subsequently, this category was revised as a trigger in “non-cesarean section patients who used laxatives or stool softeners”.

After a comprehensive validation process, the modified triggers contained a collective sum of 35 items, inclusive of 12 laboratory tests, 8 medications, 11 symptoms, and 4 outcomes.

## Research limitations

There are still some limitations to the present study. First, one ADE eluded detection through obstetric triggers due to the inherent limitations of the GTT in identifying particular categories of ADEs [78]. GTT proves ineffectual in detecting the medication errors that are rarely documented in patient records. Second, the quality of the medical records greatly biased the results, as healthcare professionals may overlook ADEs deemed subjectively inconsequential. Moreover, the demographic composition of patients and medication habits within the specific hospital under study may have constrained the positive activation of triggers [33]. Consequently, the generalizability of the findings to other healthcare institutions may be limited, necessitating tailored modifications for individualized application in varied contexts. Forth, although the study team have received identical training through a medical record examination based on the IHI white paper, there exists variability among members in the identification and assessment of ADE and their respective severity ratings, which may be attributed to limitations inherent the investigator’s clinical experience and knowledge. Haukland EC hypothesized that awareness of the outcome and its severity, a phenomenon known as hindsight bias, may have led to an overestimation of both the quantity and severity of adverse events within the inpatient death sample [41]. Last, The IHI white paper recommended a review time of 20 min for each medical record. However, in cases where medical records were complicated due to comorbidities or prolonged hospital stays, the review duration needed to be extended, potentially uncovering additional ADEs in these specific medical records.

This study still employs the manual perusal of medical records, which is inefficient and retrospective. The automated triggers facilitate the comprehensive detection of all electronic medical records, as opposed to relying on limited data samples, which holds the potential to enhance the assurance of drug safety and expedite the timely enhancement of clinical outcomes for inpatients. With the updating of guidelines and the burgeoning body of research, a periodic review and updating of the triggers featured in this study become imperative. It is noteworthy that our study merely included the literature collected in PubMed and CNKI, which introduce a potential bias, thereby impacting the overall representativeness of the findings.

## Conclusions

The obstetric triggers established in this study were proven to be more sensitive and specific in the active monitoring of ADE among obstetric inpatients compared with SRS and GTT, and provided a benchmark for ADE monitoring among obstetric inpatients within medical institutions.

## Contribution to the field statement

In this study, the GTT was employed for the inaugural monitoring of ADEs in obstetric inpatients, marking a pioneering initiative both nationwide and worldwide. By investigating the occurrence of ADR in pregnant patients in Sichuan Province, the characteristics of ADR during pregnancy were comprehensively summarized, laying the foundation for an improvement in the local suitability of triggers. According to the physiologic characteristics of pregnant patients and specific obstetric drugs administered, triggers were then revised appropriately. We considered ADEs for the female reproductive system, fetus, and newborn, and established unique obstetric triggers. We postulated that the obstetric triggers were more suitable for the practical application of inpatients in a local department, and that the triggers could predict the occurrence of ADE more efficiently and accurately than other methods.

### Electronic supplementary material

Below is the link to the electronic supplementary material.

Supplementary Material 1

Supplementary Material 2

## Data Availability

All data generated or analyzed during this study are included in this published article.

## Abbreviations

ADE: adverse drug event
ALP: alkaline phosphatase
ALT: alanine aminotransferase
APTT: activated partial thromboplastin time
BG: blood glucose
CTCAE: Common Terminology Criteria for Adverse Events
DVT: Deep venous thrombosis
FBG: fasting blood glucose
FT: Free Thyroxine
GTT: Global Trigger Tool
HELLP syndrome: hemolysis, elevated liver enzymes and low platelets syndrome
IHI: Institute for Healthcare Improvement
INR: international normalized ratio
INRUD: International Network for Rational Use of Drugs
NADRMS: National Adverse Drug Reaction Monitoring System
NC: neutrophil count
NSAIDs: non-steroidal anti-inflammatory drugs
PBG: postprandial blood glucose
PE: Pulmonary thromboembolism
PPH: Postpartum hemorrhage
PPV: positive predictive value
PT: prothrombin time
SCr: serum creatinine
SRS: spontaneous reporting system
TPOAb: thyroid peroxidase antibody
TSH: thyroid stimulating hormone
TT: Total Thyroxine
WBC: white blood cells
WHO-UMC: World Health Organization-Uppsala Monitoring Center
