# Supplementary material for: Establishing a trigger tool based on global trigger tools to identify adverse drug events in obstetric inpatients in China
Source: BMC Health Serv Res. 2024 Jan 15;24:72. doi: 10.1186/s12913-023-10449-z (PMC10789046; doi:10.1186/s12913-023-10449-z)
Supplement: Supplementary file 1 — Supplementary Material 1 [file 12913_2023_10449_MOESM1_ESM.doc]

## Medical record review sheet for applying of the obstetric trigger tool

Patient number Sex Age Delivery mode

Diagnosis Medication record

Other important information:

| **No.** | **Triggers** | **Interpretation** | **Positive trigger（yes/no）** | **ADE** |
| --- | --- | --- | --- | --- |
| L1 | K<3.3 mmol·L^-1^ | hypokalemic drugs used |  |  |
| L2 | K>5.5 mmol·L^-1^ | hyperkalemic drugs used |  |  |
| L3 | Mg>3.5 mmol·L^-1^ | hypermagnesemia drugs used |  |  |
| L4 | Na<130 mmol·L^-1^ | hyponatremic drugs used |  |  |
| L5 | Non-diabetic patients: BG <3.3 mmol/L  Diabetic patients receiving hypoglycemic therapy: BG <3.9 mmol/L | hypoglycemic drugs used inappropriately |  |  |
| L6 | Patients receiving hypoglycemic therapy: FBG >5.3 mmol/L, 1 h PBG >7.8 mmol/L, 2 h PBG >6.7 mmol/L  Non-diabetic patients: FBG ≥6.1 mmol/L, PBG ≥7.8 mmol | hyperglycemic medications used inappropriately |  |  |
| L7 | SCr increased ≥.3 mg/dL within 48 h or SCr increased to ≧1.5 times of baseline within seven days or urine volume <0.5 mL/(kg·h) & duration >6 h | nephrotoxic drugs used |  |  |
| L8 | PT >13 S; APTT >35 s; INR ≥1.5; combined with bleeding symptoms | heparin used excessively |  |  |
| L9 | Platelets <50×10^9^ /L (excluding physiologic changes and reductions caused by comorbid diseases) | thrombocytopenia drugs used |  |  |
| L10 | ①Hyperthyroidism: TSH decreases, TT4 and FT4 increase;  ②[Hypothyroidism](http://dict.youdao.com/w/hypothyroidism/#keyfrom=E2Ctranslation) TSH>4.0 mIU/L or TPOAb+ and TSH 2.5–4.0 mIU/L;Excluding combined thyroid disease | antithyroid drugs/hyperthyroidism drugs used |  |  |
| L11 | WBC Ct <5.9×10^9^/L; neutrophil count<3900/Ul (excluding decreases caused by disease changes) | leukopenia drugs used |  |  |
| L12 | ALT ≥3ULN&R ≥5, ALP ≥2ULN&R ≤2, ALT ≥3ULN, ALP ≥2ULN&2<R<5, R=(ALT measured value/ALTULN)/(ALP measured value/ALPULN) | hepatotoxic drugs used |  |  |
| M1 | protamine given | after heparin administration |  |  |
| M2 | Use of glucocorticoids/antihistamines/calcium gluconate | after drug allergy or anaphylaxis/anaphylactic shock caused by transfusion |  |  |
| M3 | Use of adrenaline | anaphylactic shock |  |  |
| M4 | 50% glucose injection (neonates 10%)administered | after drug-induced severe hypoglycemia |  |  |
| M5 | narcan (naloxone)/nalmefene | after opioid poisoning |  |  |
| M6 | laxative or stool softener given | after drug-induced constipation |  |  |
| M7 | Use of live intestinal bacteria preparations/antidiarrheal agents such as montmorillonite | after drug-induced diarrhea |  |  |
| M8 | Use of antiemetics (excluding nausea of pregnancy) | after drug-induced vomiting |  |  |
| M9 | Intravenous injection of calcium gluconate | after magnesium sulfate administration |  |  |
| S1 | Skin allergic reaction | after antibiotics/drugs that cause skin reactions administration |  |  |
| S2 | Hypotension/falls | after antihypertensive drugs, sedative hypnotics, and other drug administration |  |  |
| S3 | Elevated blood pressure: higher than systolic blood pressure of 140 mmHg and/or diastolic blood pressure of 90 mmHg (excluding poorly controlled hypertension) | after hypertensive drugs,prostaglandin drugs, ergonovine administration |  |  |
| S4 | Bleeding (including nasal bleeding, gum bleeding, gastrointestinal bleeding, skin purpura) | after drug-induced bleeding (e.g., aspirin) |  |  |
| S5 | Weak contractions, postpartum hemorrhage | after sedatives, analgesics, magnesium sulfate administration |  |  |
| S6 | Excessive uterine contractions, uterine rupture | after oxytocin, prostaglandins, ergonovine administration |  |  |
| S7 | Acral edema, facial edema, periorbital edema, pulmonary edema (the edema is not caused by the original disease) | after hormones, analgesics, NSAIDS, ergonovine administration |  |  |
| S8 | Thromboembolic events (DVT or PE) (excluding spontaneous embolism caused by pregnancy) | ①after drugs that may cause thromboembolism ②Insufficient use of anticoagulant drugs |  |  |
| S9 | Basal body temperature rise≥2°C, high fever, chills (excluding stress and infection factors) | Using drugs that increase body temperature and chills |  |  |
| S10 | Nervous system symptoms (dizziness, headache, facial or extremity numbness, lethargy, and fatigue) | after oxytocin, prostaglandins, antibiotic drugs administration |  |  |
| S11 | Gastrointestinal discomfort such as nausea and vomiting (excluding morning sickness during pregnancy) | after drugs causingadverse gastrointestinal reactions |  |  |
| S12 | Vaginal discomfort (burning sensation, pain, and local bleeding) (excluding vaginal discomfort caused by diseases such as vaginitis) | after topical vaginal medication administration |  |  |
| S13 | Heart rate higher than 140 beats/min, arrhythmia | after oxytocin, prostaglandins, ergonovine administration |  |  |
| S14 | Oligohydramnios or oligohydramnios (premature rupture of membranes excluded) | after oxytocin, prostaglandins, ergonovine administration |  |  |
| O1 | admission to ICU /rescue | ADE-induced serious illness |  |  |
| O2 | Abrupt cessation of medication (long-term use of anticoagulants, antihypertensives, hypolipidemic, hypoglycemic or hormones) | ADE caused withdrawal or ADE appeared due to withdrawal |  |  |
| O3 | Neonatal asphyxia, fetal distress, premature delivery, and neonatal respiratory depression (excluding neonatal umbilical cord torsion and other diseases) | Use of drugs that adversely affect newborns (analgesics, oxytocin, prostaglandins, and ergonovine) |  |  |
| O4 | Neonatal withdrawal symptoms (neonatal hypoglycemia, hypotension, neonatal bleeding, bradycardia, neonatal abnormal muscle movement, lethargy, severe breathing difficulties, and feeding difficulties | Use of drugs that adversely affect newborns (analgesics, oxytocin, prostaglandins, and ergonovine) |  |  |
