# Supplementary material for: Establishing a trigger tool based on global trigger tools to identify adverse drug events in obstetric inpatients in China
Source: BMC Health Serv Res. 2024 Jan 15;24:72. doi: 10.1186/s12913-023-10449-z (PMC10789046; doi:10.1186/s12913-023-10449-z)
Supplement: Supplementary file 2 — Supplementary Material 2 [file 12913_2023_10449_MOESM2_ESM.docx]

Flowchart of the study sample process

Inclusion criteria：(1) discharged medical records from July 1, 2018 to September 30, 2018; (2) hospitalized obstetric patients at a gestational age of ≥28 w; (3) a patient age of 16–65 years; and (4) a hospitalization duration of >48 h.

Exclusion criteria :(1) a lack of medication used for treatment; and (2) where the primary data of the inpatient medical record were missing.

Random biweekly sample of 50 patient records

GTT team:two trained junior pharmacists independently

Time limit:20 min/patient record

Analysis of 25 first eligible records

300 collection sheets

Trigger present

*Time 0 +45min min*

Trigger absent

Trigger positive charts reviewed by a trained pharmacist and physician to identify ADEs

End of review

No ADE identified by trigger method

No ADE Identified

ADE Identified
